# Supplementary material for: Capturing Recent Mycobacterium tuberculosis Infection by Tuberculin Skin Test vs. Interferon-Gamma Release Assay
Source: Trop Med Infect Dis. 2024 Apr 11;9(4):81. doi: 10.3390/tropicalmed9040081 (PMC11053984; doi:10.3390/tropicalmed9040081)
Supplement: Supplementary file 1 [file tropicalmed-09-00081-s001.zip › tropicalmed-2915096-supplementary.pdf]

Supporting information

Study Design

Figure S1. Study protocol for the Kampala household contact study

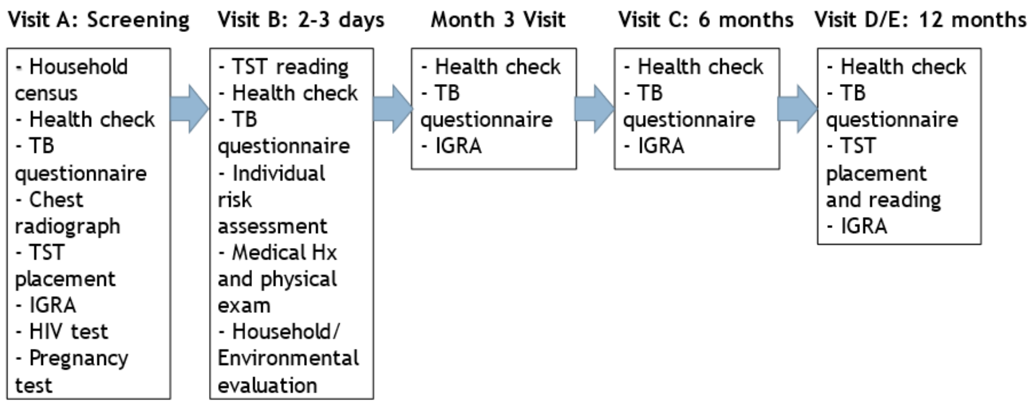

Methods

The epidemiologic risk score (ERS) is composed of the following 10 items:

- Is the index case the adult's spouse?
- Is the adult the index case's primary caregiver?
- Does the index case sleep in the same bed as the adult?
- Does the index case sleep in the same room as the adult?
- Is the index case coughing?
- Does the index case have reported pulmonary TB?
- Does the index case have smear-positive sputum?
- Does the index case live in the same household as the adult?
- Does the index case see the adult every day?
- Is there more than one adult TB case in the adult's household?

Each positive response receives one point for a total of 10 points. A higher score confers a higher risk.

Comparing the results of cluster analysis to the predetermined conversion groups

A cluster analysis of all available variables using Gower distance was performed to determine if the subject clustering obtained was similar to the predetermined conversion groups. The purpose of this analysis was to examine whether clinical and epidemiological variables could better define subgroups of HHCs and if these groupings aligned with a TST and/or QFT-based definition. For our cluster analysis, we included all HHC participants enrolled in the study by January 2022 (n=383) and all the variables included in our univariate analysis. We used Gower distance which allowed us to measure how different two HHCs were based on both numerical and categorical variables. The distance varied between 0 (identical) to 1 (maximally dissimilar). In addition, the number of predetermined clusters was set to 2 based on the results of a Silhouette analysis. Therefore, our model used partitioning around mediods (PAM) with two clusters.

This model resulted in 203 HHCs being allocated into cluster 1 and 180 HHCs being allocated into cluster 2, as shown in Supp Fig 2. When we compared the two clusters, we found that they differed in terms of ERS, muzigo living, cooking location, number of windows, relationship to index, location of sleeping, people per room, presence of index hemoptysis. These variables were, therefore, included in the logistic regression analysis.

Figure S2. Visualization of cluster model based on partitioning around mediods using two clusters.

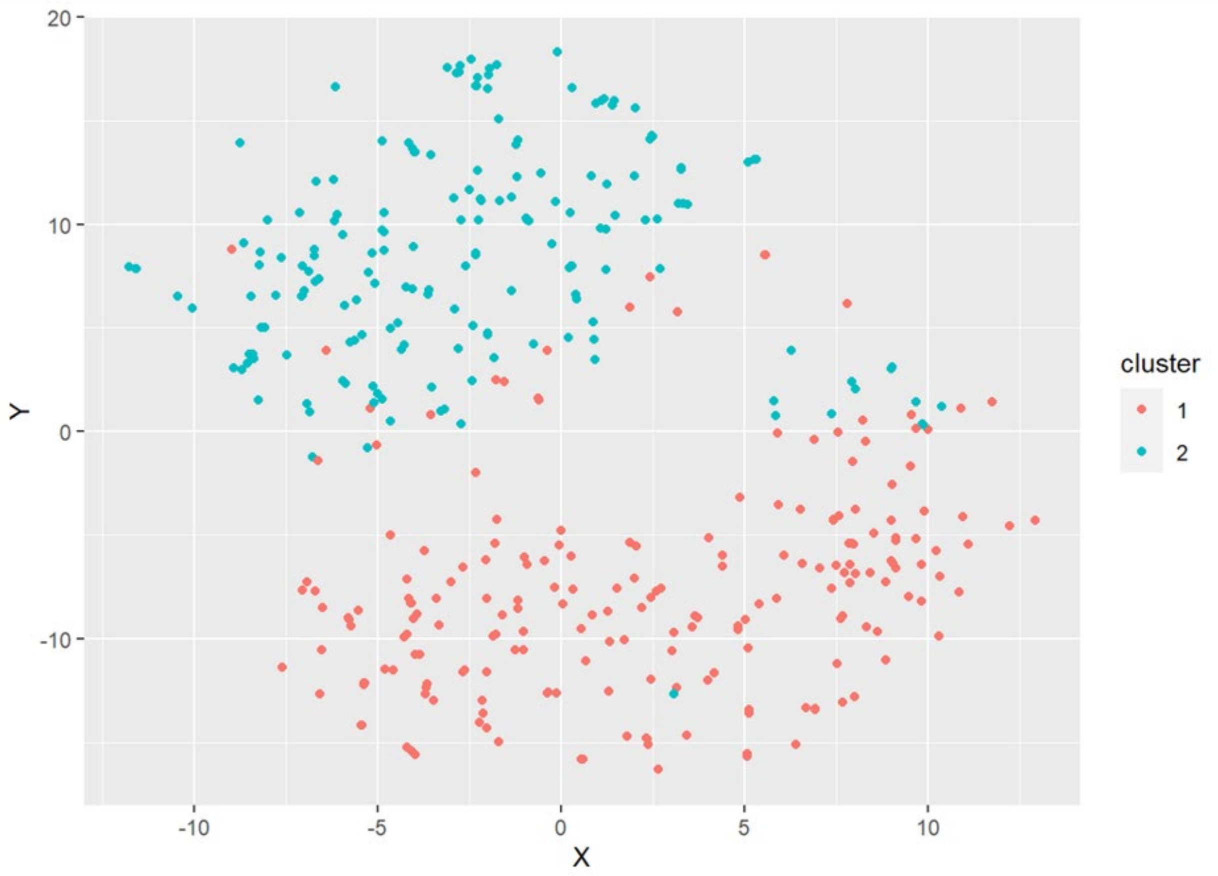

Table S1. QFT results of household contacts (HHCs) who were classified by consensus. There were 30 HHCs with indeterminate or inconsistent QFT results who were classified by consensus into one of these categories based on the preponderance of the data, when possible (S1 Table). After this process, 8 HHCs remained unclassifiable, 10 HHCs were classified as resisters, 8 HHCs were classified as LTBI, and 4 HHCs were classified as “reverters” (a HHC with a positive QFT result followed by a negative QFT result). None of these HHCs were classified as either TST or QFT converters. Otherwise, these HHCs were excluded from the statistical analyses described below. There were also 5 individuals with one positive QFT in the middle of the observation period who were excluded from the analysis. Based on a single QFT result, it is unclear if these reflect a conversion event vs. a false-positive QFT. Serial and consistent QFT results are required to reliably detect definite conversion events, i.e., recent *Mtb* infection. Variability in QFT responses over time has been observed in another study [28].

HHC	BASELINE	MONTH 3	MONTH 6	MONTH 12	QFT CLASSIFICATION
1	QFT-	NA	QFT+	QFT-	RESISTER
2	QFT+	NA	QFT+	QFT-	POSSIBLE REVERTER
3	QFT+	NA	QFT+	QFT-	POSSIBLE REVERTER
4	QFT-	NA	QFT-	INDETERMINATE	RESISTER
5	INDETERMINATE	NA	QFT+	QFT+	UNINTERPRETABLE
6	QFT+	NA	QFT-	QFT+	LTBI
7	QFT+	NA	INDETERMINATE	QFT+	LTBI
8	QFT-	NA	QFT+	QFT-	POSSIBLE RESISTER
9	QFT+	NA	QFT+	QFT-	POSSIBLE REVERTER
10	QFT+	NA	QFT+	QFT-	POSSIBLE REVERTER
11	INDETERMINATE	NA	QFT-	QFT-	RESISTER
12	QFT-	NA	QFT+	QFT-	UNINTERPRETABLE
13	QFT-	NA	QFT+	QFT-	UNINTERPRETABLE
14	QFT-	NA	QFT+	QFT-	UNINTERPRETABLE
15	QFT+	NA	QFT+	QFT-	UNINTERPRETABLE
16	QFT-	QFT+	QFT-	QFT+	UNINTERPRETABLE
17	QFT-	QFT+	QFT-	MISSED VISIT	POSSIBLE RESISTER
18	QFT+	QFT+	QFT+	QFT-	LTBI
19	QFT+	QFT+	QFT-	QFT+	LTBI
20	QFT-	QFT-	QFT+	QFT-	POSSIBLE RESISTER
21	QFT+	INDETERMINATE	INDETERMINATE	MISSED VISIT	UNINTERPRETABLE
22	QFT+	QFT-	QFT+	QFT-	POSSIBLE RESISTER
23	QFT-	QFT-	QFT+	QFT-	POSSIBLE RESISTER
24	QFT+	QFT-	QFT+	QFT+	LTBI
25	QFT-	QFT-	QFT+	QFT-	POSSIBLE RESISTER

26	QFT-	QFT-	MISSED VISIT	INDETERMINATE	RESISTER
27	QFT+	QFT+	QFT+	QFT-	LTBI
28	QFT+	QFT-	QFT-	QFT+	UNINTERPRETABLE
29	QFT+	INDETERMINATE	QFT+	MISSED VISIT	LTBI
30	QFT+	INDETERMINATE	INDETERMINATE	QFT+	LTBI

Table S2. Comparing household contacts included in the analysis versus those who were excluded. The household contacts that were excluded had a higher proportion of female participants and a higher TB risk score.

	**Included**	**Excluded**	***p*-value (test)**
**N**	**148**	**69**	**NA**
**Age**	**25 [20-40.3]**	**27 [20-37]**	**0.78 (KW test)**
**Sex (female)**	**91 (61.5%)**	**52 (75.4%)**	**0.047* (Fisher's)**
**HIV (positive)**	**10 (6.8%)**	**6 (8.7%)**	**0.59 (Fisher's)**
**BCG scar present**	**110 (74.3%)**	**49 (71%)**	**0.51 (Fisher's)**
**TB risk score**	**6 [6-7]**	**7 [6-8]**	**0.02* (KW test)**
**Living in Muzigo**	**59 (39.9%)**	**34 (49.3%)**	**0.24 (Fisher's)**

Counts (percentages) or median [quartiles]
KW: Kruskal-Wallis
*** Statistically significant at *p*<0.05**

Table S3. Univariate analysis comparing QFT-only, TST-only and QFT/TST converters based on individual and household characteristics. For this comparison, QFT-only converters and QFT/TST converters were combined into a single category, QFT converters.

	**TST-only converters**	**QFT converters**	**"Resisters"**	***p*-value (test)**	**Relevant pairwise *p*-value**
N	39	37	72	NA	NA
**Individual Characteristics**					
Age	32 [20-47]	26 [21-38]	23 [19.8-36.5]	0.11 (KW test)	TST-only vs. "Resister": 0.03*
Sex (female)	21 (53.8%)	27 (73.0%)	43 (59.7%)	0.20 (Fisher's)	NA

BCG scar present	31 (81.6%)	26 (70.3%)	53 (75.7%)	0.48 (Fisher's)	NA
HIV positive	3 (7.7%)	3 (8.1%)	4 (5.6%)	0.76 (Fisher's)	NA
BMI	23.6 [19.5-27.0]	22.2 [20.2-28.1]	22.8 [21.0-25.2]	0.91 (KW test)	NA
TB Risk score	6 [6-7]	7 [6-8]	6 [6-7]	0.14 (KW test)	NA
Quantitative IGRA values at conversion	3.4 [0.3-9.3]	3.5 [1.6-8.4]	NA	0.48 (MWU test)	NA
No history of smoking	37 (94.9%)	35 (94.6%)	61 (84.7%)	0.20 (Fisher's)	NA
Spouse to Index	7 (17.9%)	9 (24.3%)	8 (11.1%)	0.13 (X^2)	NA
**Household Characteristics**					
Living in Muzigo	20 (51.3%)	18 (48.6%)	21 (29.2%)	0.03* (Fisher's)	QFT-comb vs. "Resister": 0.05*<br>TST-only vs. "Resister": 0.02*
Cooking inside home	10 (25.6%)	12 (32.4%)	28 (38.9%)	0.38 (Fisher's)	NA
Number of windows	1 [1-3]	2 [1-3]	3 [1-4]	0.19 (KW test)	NA
People per room	2 [1.33-3]	2 [1-3]	2 [1.3-2.67]	0.36(KW test)	NA
Sleeping in same room	21 (53.8%)	26 (70.3%)	33 (45.8%)	0.053 (X^2)	NA
Sleeping in same bed	6 (15.4%)	11 (29.7%)	12 (16.7%)	0.20 (X^2)	NA

Counts (percentages) or median [quartiles]
ERS: Epidemiologic risk score
KW: Kruskal-Wallis
MWU: Mann–Whitney *U* test
X^2: Chi-square test
* Statistically significant at $p<0.05$
¶ Statistically significant after Bonferroni correction at $p<0.006$
# Statistically significant after Bonferroni correction at $p<0.008$

Table S4. Univariate analysis comparing QFT-only, TST-only and QFT/TST converters based on index case characteristics. For this comparison, QFT-only converters and QFT/TST converters were combined into a single category, QFT converters.

	**TST-only converters**	**QFT converters**	**"Resisters"**	***p*-value (test)**	**Relevant pairwise *p*-value**
Sex (female)	20 (51.3%)	25 (67.6%)	35 (48.6%)	0.16 (Fisher's)	NA
BCG scar present	30 (76.9%)	27 (75%)	36 (56.3%)	0.06 (Fisher's)	NA
HIV positive	4 (12.9%)	1 (3.2%)	12 (18.8%)	0.11 (Fisher's)	NA
BMI	18.5 [17-21]	19 [17-20]	19 [18-20]	0.45 (KW test)	NA
Cavitary lesions present	32 (82.1%)	33 (89.2%)	43 (60.6%)	0.002* (Fisher's)	TST-only vs. "Resister": 0.04* QFT comb vs. "Resister": 0.004*
Advanced lung disease	24 (61.5%)	17 (45.9%)	25 (35.2%)	0.03* (Fisher's)	TST-only vs. "Resister": 0.01*
Positive Smear	29 (100%)	28 (96.6%)	55 (100%)	0.51 (Fisher's)	NA
Positive GeneXpert	39 (100%)	37 (100%)	71 (98.6%)	1.00 (Fisher's)	NA
Coughing	39 (100%)	37 (100%)	71 (100%)	1.00 (Fisher's)	NA
Cough duration (days)	90 [60-142.5]	60 [30-75]	60 [30-90]	0.01* (KW test)	TST-only vs. QFT comb: 0.005* TST-only vs. "Resister": 0.017*
Fever	30 (76.9%)	28 (75.7%)	54 (76.1%)	0.99 (X^2)	NA
Fever duration (days)	60 [30-90]	30 [21-60]	30 [14-56.3]	0.05* (KW test)	TST-only vs. "Resister": 0.02*
Productive sputum	36 (92.3%)	37 (100%)	68 (95.8%)	0.32 (Fisher's)	NA

Productive sputum duration (days)	60 [30-120]	30 [30-60]	30 [30-60]	0.06 (KW test)	NA
Purulent sputum	22 (56.4%)	20 (54.1%)	37 (52.9%)	0.94 (X^2)	NA
Purulent sputum duration (days)	52.5 [30-120]	30 [30-60]	30 [15-90]	0.16 (KW test)	NA
Hemoptysis	4 (10.3%)	9 (24.3%)	7 (10.0%)	0.12 (Fisher's)	NA
Hemoptysis duration (days)	5 [2.5-12.8]	2.5 [1.75-8.75]	7 [2.5-10.5]	0.88 (KW test)	NA
Dyspnea	20 (52.6%)	16 (43.2%)	38 (53.5%)	0.59 (Fisher's)	NA
Dyspnea duration (days)	90 [30-160]	60 [30-90]	30 [30-82.5]	0.09 (KW test)	NA
Weight loss	33 (84.6%)	30 (81%)	59 (83.1%)	0.92 (Fisher's)	NA
Weight loss duration (days)	60 [30-90]	60 [30-75]	30 [30-82.5]	0.10 (KW test)	NA

Counts (percentages) or median [quartiles]
ERS: Epidemiologic risk score
KW: Kruskal-Wallis
X^2: Chi-square test
* Statistically significant at $p<0.05$
¶ Statistically significant after Bonferroni correction at $p<0.002$
